# Pido: Predictive Delay Optimization for Intertidal Wireless Sensor Networks

**DOI:** 10.3390/s18051464

**Published:** 2018-05-08

**Authors:** Xinyan Zhou, Xiaoyu Ji, Bin Wang, Yushi Cheng, Zhuoran Ma, Francis Choi, Brian Helmuth, Wenyuan Xu

**Affiliations:** 1 College of Electrical Engineering, Zhejiang University, Hangzhou 310027, China; xinyanzhou@zju.edu.cn (X.Z.); yushicheng@zju.edu.cn (Y.C.); mzr@zju.edu.cn (Z.M.); wyxu@zju.edu.cn (W.X.); 2 Hangzhou Hikvision Digital Technology Co Ltd., 310051 Hangzhou, China; wangbin2@hikvision.com; 3 Department of Marine and Environmental Sciences and School of Public Policy and Urban Affairs, Northeastern University Marine Science Center, Nahant, MA 01908, USA; f.choi@northeastern.edu (F.C.); b.helmuth@northeastern.edu (B.H.)

**Keywords:** delay modeling, delay optimization, intertidal WSN, environmental monitoring, ETX

## Abstract

Intertidal habitats are among the harshest environments on the planet, and have emerged as a model system for exploring the ecological impacts of global climate change. Deploying reliable instrumentation to measure environmental conditions such as temperature is challenging in this environment. The application of wireless sensor networks (WSNs) shows considerable promise as a means of optimizing continuous data collection, but poor link quality and unstable connections between nodes, caused by harsh physical environmental conditions, bring about a delay problem. In this paper, we model and analyze the components of delays in an intertidal wireless sensor network system (IT-WSN). We show that, by properly selecting routing pathways, it is feasible to improve delay. To this end, we propose a Predictive Delay Optimization (Pido) framework, which provides a new metric for routing path selection. Pido incorporates delay introduced by both link quality and node conditions, and designs a classifier to predict future conditions of nodes, i.e., the likely time of aerial exposure at low tide in this case. We evaluate the performance of Pido in both a real IT-WSN system and a large-scale simulation, the result demonstrates that Pido decreases up to 73% of delays on average with limited overhead.

## 1. Introduction

The intertidal zone, i.e., the region between the low and high tide lines, serves as a key testbed for exploring the effects of climate change on the distribution and abundance of organisms [1]. Environmental conditions in this habitat are among the harshest on Earth: waves frequently exert forces equivalent to those produced by wind speeds of over 1000 km h^−1^ on land, and organisms are exposed to highly fluctuating levels of temperature, salinity and pH. Moreover, intertidal organisms (e.g., mussels, seastars, kelps) are exposed to the terrestrial environment each day at low tide for periods of up to 6–8 h or more. As ectothermic organisms, these plants and animals have body temperatures that are driven primarily by exposure to solar radiation [2], thus fluctuations in animal temperature of 25 °C or more over the course of a few hours are common.

Recent studies have begun to pair local records of temperatures relevant to intertidal organisms [3] with controlled laboratory experiments to better understand and predict climate change impacts on this model system. However, making continuous recordings of temperatures is extremely difficult, especially in real time given high rates of instrument loss and failure. The development of a robust sensor network has shown considerable promise as a mechanism for addressing a host of ecological problems [4,5,6].

We deployed intertidal wireless sensor network systems (IT-WSN), at intertidal sites in Boston, MA and Georgetown SC (USA); and Hong Kong and Zhoushan (China). Our experimental results based on field tests demonstrate that WSNs in intertidal zones suffer several unique challenges, such as extremely unstable link qualities, intolerable delays and low packet delivery ratios. For example, it can take hours or even days to deliver a packet in IT-WSNs when the sensors are submerged under-water, which is far longer than that in common WSNs for other environments. Furthermore, we found that the buffer condition also played as an important role in the delay problem. This paper addresses the issue of reducing end-to-end packet delivery delays in intertidal WSNs.

After analyzing the data we collected from IT-WSNs, we find that, not surprisingly, the changeable node status (i.e., available or not available) and unstable link quality caused by the wave force are the main reasons for the long delay. We show that existing delay optimization protocols such as DutyCon (Dynamic duty cycle Control scheme) [7], EDAL (Energy-efficient Delay-Aware Lifetime balancing data collection protocol) [8], BCMN/A (Broadcasting Combined with Multi-NACK/ACK) [9] and ORW (Opportunistic Routing in WSNs) [10] used to minimize packet delivery delay are not applicable for IT-WSNs, as these protocols focus on selecting a routing path with less transmission delay in relatively stable networks. In contrast, routing protocols like CREST [11] and CSPR (Conditional Shortest Path Routing) [12] are specifically designed for intermittent WSNs, which have similar characteristics with IT-WSNs. However, these protocols fail to consider the node conditions, e.g., the node buffer and node availability, which may lead to buffer overflow and unnecessary delay. Thus, the delay problem for IT-WSNs is yet to be solved.

In the IT-WSN, the key difficulties for delay optimization are (a) to predict a future link quality of a node, i.e., whether a node is available or how good the quality of a link is, and (b) to consider the dynamics of nodes and links caused by changes in the environment. With this in mind, we attempted to reduce the delay in IT-WSNs by designing a suitable routing metric. With the new routing metric, nodes can predictively choose routes with less delay. To this end, we propose Pido, a Predictive delay optimization framework for intertidal WSNs.

The design of Pido faces several challenges. First, the sources of delays in the IT-WSN are complex and we need to take all delay components into account for delay metric design. After analyzing the delays in real IT-WSNs, we find extreme dynamic link quality, occupied buffer condition of nodes and fierce sea wave in the intertidal zone are the causes of delay problem. Combining delays from above sources and quantifying them accurately are crucial yet challenging. Second, it is challenging to predict future status of nodes and links accurately, even if obtaining the current status of nodes. While tidal predictions offer some prospect of predicting how long the delay will last based on shore elevation, the presence of waves and winds can make these predictions complicated. The disconnection and reconnection between nodes show imprecise statistical patterns, and therefore cannot be utilized for accurate prediction.

To overcome the above challenges, we first model the delay in consistence IT-WSN. We divide delays into (a) queuing delay caused by intermittent connections, (b) queuing delay caused by buffer occupancy and (c) transmission delay caused by poor link quality, and we further summarize them into node delay and link delay. For prediction of node status, we utilize three implicit indicators instead of directly forecasting the future status of nodes. With the indirect indicators, we train a lightweight classifier to achieve accurate node status prediction. We extensively verify the feasibility of predicting node future status with field test results we collected in the IT-WSN deployed in Zhoushan, China. We further evaluate the performance of Pido in a large-scale simulation, and results indicate that Pido can decrease system end-to-end delay effectively with limited overhead on energy consumption.

The remainder of the paper is organized as follows. We first discuss the related works and summarize our contributions in Section 2. We then explain the motivation and the application scenario of our work, present the design of Pido in details and evaluate its performance in Section 3. We then provide an overall assessment of Pido in Section 4.4 and conclude this paper in Section 6.

## 2. Related Works and Contributions

The related work can be classified into three categories: link estimation metrics, routing protocols in the WSN and delay optimization solutions.

Choosing the path with good “quality” for packets delivering is fundamental for routing protocols in WSNs. Different requirements in applications have variant definitions about good quality. During the past decades, significant research has been conducted to estimate quality on different dimensions. Hardware based estimation methods select RSSI (received signal strength indicator) [13], SNR (signal noise ratio) [14] and LQI (link quality indicator) [15] as important indicators of link quality. Utilizing hardware based indicators is overhead free; however, the measurement of RSSI cannot reflect the condition of link quality in real time and may fail to select the best path in real systems. Software based link quality methods like CTP (collection tree protocol) [16] and 4-bit [17] search the path with least retransmission times in real time. CTP [16] exploits link quality by calculating packet delivery ratio bi-directionally, and 4-bit [17] provides a comprehensive method by combining quality of link layer, network layer and physical layer. These two methods are user friendly on implementation and wildly used in real WSNs. Besides CTP, other link quality metrics like ETOP (Expected number of Transmissions On a Path) [18] and L-NT in [19] are proposed to select route wisely. However, these link metrics emphasize link connection individually and do not consider the situation of nodes, which may not fit in the requirement of IT-WSNs.

Besides link quality, energy-awareness becomes another important requirement in most WSNs. Sensors in WSNs are battery-powered, which makes energy become a valuable resource, and routing protocols such as EAP (Energy-Aware Routing) [20] and REAR (Reliable Energy Aware Routing) [21] are proposed to provide energy-efficient routing services. EAP provides a new clustering parameter for cluster head election and can balance the energy load among all nodes. REAR provides a reliable transmission environment for data delivery in an energy efficient way by introducing local node selection, path reservation and path request broadcasting delay. These routing protocols are focusing on energy consumption optimization. In IT-WSN, nodes consume less energy during the under-water phase. Users care more about the real-time performance, which cannot be solved with EAP and REAR.

Delay optimization solutions are proposed to decrease system latency. For example, EDAL (Energy-efficient Delay-Aware Lifetime balancing data collection protocol) [8] generates routes with minimal total path cost among all source nodes, under the constraints of delay requirements. The global optimization mechanism in EDAL is not suited for intertidal environments, where nodes in the IT-WSN face variant routing problems. BCMN/A (Broadcasting Combined with Multi-NACK/ACK) [9] proposes a mechanism to reduce the energy consumption and delays during the broadcast procedure. In the IT-WSN, we focus on reducing the end-to-end delay of data transmission, and BCMN/A is inapplicable. Landsiedel et al. [10] proposed ORW (Opportunistic Routing in WSNs), which introduces EDC (Expected Duty Cycled wakeups) as a metric for routing selection. However, the delay caused by the node condition is not considered in ORW, which may not suit for the intertidal WSN.

There are also several delay-constrained routing protocols specially designed for intermittent WSNs. DutyCon (Dynamic duty cycle Control scheme) [7] introduces a self-adaptive duty cycle control approach and decomposes end-to-end delay into single-hop parts based on well-established feedback control theory. BCP (Backpressure Collection protocol) [22] presents a dynamic backpressure routing in wireless networks and uses floating LIFO (last-in-first-out) queues to decrease the end-to-end delay. However, both of these approaches focus on asynchronous networks, and do not consider the intermittent characteristics of link connection. Delay-oriented routing protocols in intermittent networks like CREST [11] and CSPR [12] are designed for intermittent situations. CREST [11] uses conditional residual time to opportunistically forward message and CSPR [12] routes message over conditional shortest path. These two protocols working in intermittent networks require the stable patterns, while, in IT-WSNs, the intermittent pattern is not stable enough and may lead to estimation error with CREST and CSPR. On the other hand, CREST and CSPR do not consider buffer condition, and may not work well in IT-WSN.

Different from the above mechanisms, Pido is designed for intertidal WSNs and provides delay optimization routing paths. We summarize our contributions in this paper as follows:

- We deploy a wireless sensor network in the intertidal area in Zhoushan and systematically investigate the components of delays based on real traces collected from the IT-WSN. Due to the harsh deployment environment, delays in IT-WSNs can be dramatically longer than WSNs more commonly used in terrestrial environments. To the best of our knowledge, this is the first work on delay optimization in real IT-WSNs.
- We analyze the feasibility of utilizing implicit indicators to predict nodes future status for IT-WSNs. We specially select indicators and compare the prediction accuracy among different classification algorithms.
- We introduce Pido , a delay optimization framework for IT-WSNs. Pido jointly considers delays caused by link quality and node conditions.
- We evaluate the performance of Pido with real-world system data and implement it in a large-scale simulation with various network sizes. We compare the results with CTP (Collection Tree Protocol) and ORW (Opportunistic Routing in WSNs), and exhaustive results demonstrate that Pido can optimize the system delay with limited overhead.

## 3. System Deployment and Motivation

In this section, we first investigate delay problems in an intertidal WSN, and then we propose a possible solution for reducing delays in such a WSN system.

### 3.1. Intertidal WSN

Intertidal zones have long served as a model system for investigating the impacts of environmental conditions such as temperature on the ecology of plants and animals [23]. Largely due to the rhythm of the tidal period, organisms in this habitat (barnacles, seaweeds, mussels, oysters, seastars, etc.), which evolved in purely marine conditions, are exposed to the terrestrial environment at low tide. In an era of rapid climate change, the intertidal zone has emerged as a model for experimentally investigating the impacts of climate change over a range of scales [24]. An important approach is comparing the current and future conditions experienced by organisms in the field to their physiological limitations, measured in the lab. What makes such an approach so difficult is that environmental conditions experienced by animals are hyperlocal and have extremely high temporal and spatial variability. For example, except for mammals and birds, the body temperatures of the vast majority of plants and animals living in air is driven by exposure to solar radiation. Body temperatures in most environments are often considerably higher than air temperature, and depending on shading animals located only a few cm apart can differ in their temperatures by >15 °C . Body temperature and physiological stress cannot, therefore, be predicted from metrics such as air temperature [24] and the only recourse is to make direct measurements at the level of the organisms using biomimetic sensors that mimic the thermal characteristics of the animal of interest [25].

Intertidal habitats make this challenging for several reasons. First, the constant action of waves make the use of any cables or wires problematic for more than a few hours. Second, because sensors need to match the spatial scale of the organism of interest, they need to be small, despite the requirement that they be encased in a waterproof housing. This challenge has been previously solved using biomimetics based on commercial loggers that are semi-permanently attached to rock substrate and left in place for periods of up to nine months, after which time they are retrieved [3,26]. While effective, this method has several problems. First, instrument losses are high, and especially in remote locations instrument loss is not noted until the end of the deployment. Second, when loggers are lost, so are all of the data. Finally, modern methods of climate adaptation are turning increasingly towards early warning systems, which requires real-time access to field data, and frequently from remote locations. Wireless networks have been used in terrestrial systems to circumvent similar challenges. For example, wildlife such as zebras fitted with radio collars that exchange location data when in proximity to one another are an effective means of ensuring that data can be retrieved even when animals are eaten by predators [27]. However, these solutions have not been adapted to intertidal systems.

**Figure 1:**
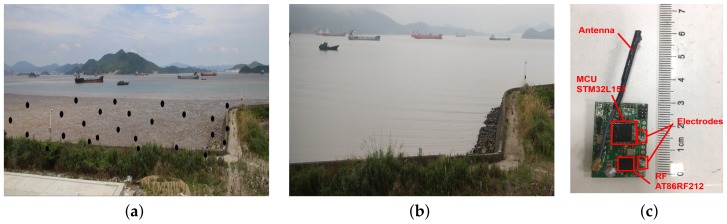
The illustration of the intertidal wireless sensor network in Zhoushan. (**a**) Nodes deployment with low tide in Zhoushan; (**b**) High tide in Zhoushan, all nodes are submerged; (**c**) The hardware design of the sensor node.

We deployed a wireless sensor network in a tidal flat in Zhoushan, China (N 29°56′43″, E 122°5′10″), which is shown in Figure 1. Figure 1a,b reveal the low-tide and high-tide of the tidal flat, and 26 nodes (which appear as black dots) are deployed for temperature monitoring. Nodes in the system cover an area of over 45,000 m^2^, and the average distance between nodes is 20 m. To fix the node in the tidal flat, we tie sensors on wooden sticks that are sunk into the mud. Tides in this area are semidiurnal, and nodes are submerged by water for over 12 h per day.

Figure 1c reveals the hardware design of the sensor node. Nodes are driven by a micro controller STM32L151 operating at 915 MHz. A pair of electrodes are equipped to acquire the current status of each node: above water or underwater. The voltage difference between electrodes will drop to zero when nodes are submerged in water. The theoretical maximum transmission range is around 300 m with a transmission power of −10 dBm. However, the PDR (packet delivery ratio) drops severely when the distance between nodes is over 100 m according to our test (the PDR is less than 20% when the transmission distance is 150 m). To deploy sensor nodes in the intertidal area, we cover the sensor node (except electrodes) with transparent epoxy (not shown in the figure) for waterproofing.

We also implement the software design of sensor nodes with CSMA (carrier sense multiple access) as MAC (multiple access control) protocol and CTP as a routing protocol. The IT-WSN is a self-organized multi-hop network, and nodes are synchronized with the base station, which is located on dry land. For each minute, nodes are operated in the active mode for the first second to sense the environment and communicate with neighbors, then they turn to the sleep mode (low power mode) for the rest of the 59 s for energy efficiency. The system cycle (System cycle is the system operating cycle and we set the system cycle as 1 min in our system. We refer to system cycle as “cycle” in the remaining of the pages.) is 1 min. The base station has infinite resources for power and buffer space. During the running procedure, nodes can read current status (above or under water) by reading the voltage difference between electrodes in Figure 1c.

### 3.2. Delay Problem in IT-WSN

**Figure 2:**
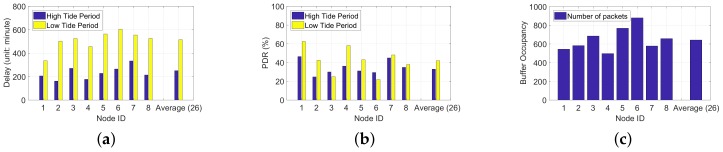
The delay, PDR and buffer condition in an IT-WSN in Zhoushan, China. We evaluate the average delay and PDR of all 26 nodes (“Average (26)”) in the Zhoushan system, and we select eight nodes (Node 1–Node 8) as representatives. (**a**) The average delay of 8 nodes and the average delay of all nodes in Zhoushan experiment during high tide period and low tide period; (**b**) The average PDR of 8 nodes and the average PDR of all nodes in Zhoushan experiment during high tide period and low tide period; (**c**) The average buffer occupancy of 8 nodes and the average buffer occupancy of all nodes in Zhoushan experiment.

Unlike other WSN systems such as [28,29], the communication in the IT-WSN is greatly impacted by environment. For example, radio signals in water strongly attenuate, creating asymmetric communication links, and the changes of high tide and low tide have tremendous effect on system performance as well, especially on end-to-end delay and PDR. Figure 2a,b provide an overview of the statistical delay and PDR in the IT-WSN. We evaluate the average delay and PDR of all 26 nodes in the Zhoushan system, and we select eight nodes who cover a wide area with different altitudes as representatives. It is worth mentioning that we count delays in system cycles, which means if a packet is generated at n-th cycle and received by the sink at m-th cycle, the end-to-end delay of this packet is (m−n) cycles. We classify delays in IT-WSNs into three categories: queuing delay caused by intermittent connections, queuing delay caused by buffer occupancy, and transmission delay caused by poor link quality.

**Queuing delay caused by intermittent connections.** Nodes are submerged by sea water for hours during the high tide period, and when nodes are under-water, the wireless channel is unavailable due to signal attenuation. The signal attenuation can be calculated by $\alpha =0.0173\times \sqrt{f\times \sigma }$, where *f* is the radio frequency in Hertz, and σ is the conductivity of the seawater (typically 4 mhos/m). Consider a sensor node operating at 916 MHz, the attenuation under-water is 1046 dB/m, which results in a communication range of less than 12 cm at a transmission power of 10 dBm. Based on this, nodes can only send packets during the low tide period. Nodes are submerged during the high tide period, and packets can only be sent until the high tide period comes, and an extremely large delay is introduced. Though delay caused by intermittent connections cannot be avoided in the IT-WSN, it is possible to decrease it by choosing a better route considering the availability of nodes.

**Queuing delay caused by buffer occupancy.** Nodes store packets locally during the under-water phase, thus buffers are strongly possible occupied. Together with the poor link quality and accumulated packets, newly arrived packets have to wait for a long time to be sent. Figure 2c shows the average buffer occupations of all nodes and eight representative nodes in the Zhoushan system, and reveals that buffers are heavily occupied. The heavy buffer occupancy also brings about packet drop as shown in Figure 2b and severely impacts the system performance. Therefore, buffer condition should be considered for delay optimization.

**Figure 3:**
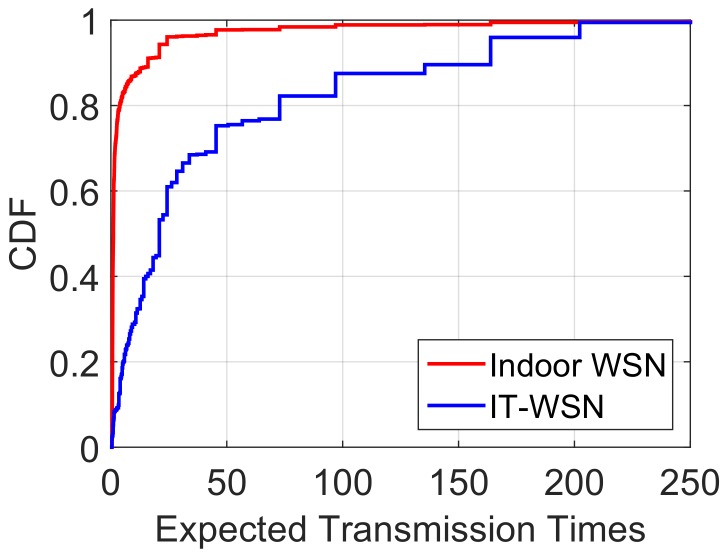
The expected transmission times distribution in an indoor WSN and an IT-WSN in Zhoushan.

**Transmission delay caused by poor link quality.** During the above-water period, the IT-WSN is supposed to be similar in stability and efficiency to a normal WSN. Unfortunately, the link quality of the IT-WSN is much worse than that in stable WSNs (e.g., terrestrial WSNs), and leads to non-negligible delays. To have a deep understanding of the link quality in the IT-WSN, we compare the ETX (expected transmissions) of routes in an indoor WSN and in the Zhoushan system. ETX proposed in CTP [16] measures the quality of a path, and the value of ETX reveals the expected times of transmissions of a packet from the source node to the destination node. A smaller value of the ETX represents a routing path with less cost. To obtain a similar deployment environment, we deploy 26 nodes in the indoor WSN, which work in the same operating mode with nodes in the Zhoushan system, and the distances between them vary from 3 m to 30 m. Figure 3 revels the CDF (cumulative distribution function) of the ETX in the indoor WSN and the IT-WSN, which represents the distribution of the ETX among all links. Results shown in Figure 3 indicate that the ETXs of over 80% of links are fewer than five for the indoor WSN, and the value is 75 for the IT-WSN case. Considering that each system has a retransmission limit, over 80% of packets in the indoor WSN can be sent in a single cycle while an IT-WSN needs 15 cycles given a retransmission limit of five cycles. Hence, poor link quality leads to extra transmission cycles, which in turn causes extra delays (14 cycles in this case).

### 3.3. Predicting Node Status

After verifying the delay components in IT-WSNs, we find that the key to optimizing delay is to identify node status, e.g., node availability and its link quality, in advance. As described above, predicting the status when nodes will be aerially exposed is difficult. By analyzing the running process of nodes in the Zhoushan system, we identify several metrics that are related to the node status. For example, during the high tide period, the constant submersion impacts link quality between nodes, especially for those deployed lower on the shore that are submerged first. Thus, a sudden change of link quality and the number of neighbors may reveal the change of water level to a certain extent. These characteristics can be well-organized and utilized as good indicators for node status prediction.

In summary, IT-WSNs suffer from queuing delay due to the intermittent connections and buffer condition, and non-ignorable transmission delay caused by the poor link quality. Considering these factors comprehensively, we design Pido to optimize delays for IT-WSNs. In the following sections, we first provide an overview of our design (Section 4) and then describe modeling in detail in Section 4.2.

## 4. Design of Pido

Combining node future status and dynamic link quality, we design Pido , which provides a new metric for route selection. In this section, we first investigate inherent characteristics in IT-WSNs and then profile Pido in general.

### 4.1. Overview of Pido

Pido utilizes delays as metric to select routing paths. There are three types of delays in IT-WSNs as explained in Section 3: (1) queuing delay caused by intermittent connections, (2) queuing delay caused by buffer occupancy and (3) transmission delay caused by poor link quality in Section 3. We further summarize delays into node delay (nd) and link delay (ld) in Pido . Node delay ($nd_{j}$) is the additional delay introduced by node *j* individually, and is related to the process capability, buffer condition and future status of node *j*. The link delay ($ld_{ij}$) is related to the link quality from node *i* to node *j*, which reveals the delay cost of transmitting a packet from node *i* to node *j*. We will describe the detail of calculating link delay (ld) and node delay (nd) in Section 4.2.

**Figure 4:**
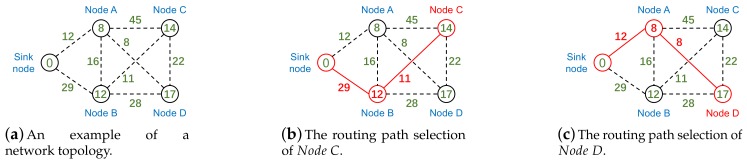
(**a**) the numbers in circles represent the node delays and the numbers on dash lines are link delays; (**b**) the routing path for NodeC is: NodeC->NodeB->Sinknode, and the overall delay is 52 cycles; (**c**) the routing path for NodeD is: NodeD -> NodeA -> Sinknode, and the overall delay is 28 cycles.

Pido selects the routing path with the least end-to-end delay and Figure 4 is an example of routing selection in Pido . Figure 4a is a typical network with four nodes and a sink node, and each node attempts to select a routing path to the sink node with the least end-to-end delay. The numbers in circles represent the node delays and the numbers on dash lines are link delays. Figure 4b,c reveal the routing paths for NodeC and NodeD. Both NodeC and NodeD have three candidate parents, and they select NodeB and NodeA as parents individually, which have the least end-to-end delay.

**Figure 5:**
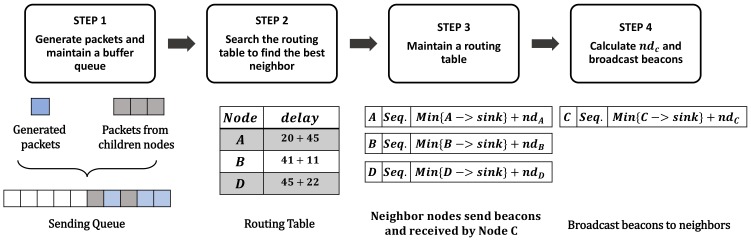
The routing selection procedure of NodeC.

Pido helps nodes to select parents with least end-to-end delays and Figure 5 reveals the mechanism of Pido . We take NodeC in Figure 4 as an example.

- **Step 1:** The node generates a packet at each cycle, and receives packets from children. Packets are stored in a sending queue and sent based on the FIFO (first in first out) rule.
- **Step 2:** When the sending queue is not empty, the node checks the routing table and searches for the best candidate to forward packets.
- **Step 3:** The node then maintains a routing table and updates it with beacons from neighbors.
- **Step 4:** The node activates self-check to calculate the process capability and buffer status, and broadcasts beacons periodically to announce the delay from itself to the sink ($Min\left\{C->sink\right\}+nd_{C}\left(t\right)$) to neighbors.

Broadcasting beacons can help neighbors to update their routing tables, and the detail of this mechanism is similar with CTP, which can be searched in [16]. Notably, when the buffer is nearly full, NodeC then alarms neighbors not to send packets to it by sending beacons with infinite delay. In this way, a node can solve the problem of packet drop caused by overflow effectively in a lightweight method.

### 4.2. Delay Modeling in Intertidal WSN

Pido always selects the routing path with the least end-to-end delay among all available paths. For a routing path with *n* hops, the end-to-end delay can be represented as follows:

(1) $$delay=\sum_{i=1}^{n}ld_{i,i+1}+\sum_{j=2}^{n-1}nd_{j+1}.$$

Before we describe how to calculate the link delay (ld) and the node delay (nd) in Equation (1), we summarize our notations in Table 1.

#### Modeling of Link Delay and Node Delay

In the IT-WSN, the poor link quality and dynamic tidal water lead to great delays, and the burstiness of packets makes the situation much worse. To better quantify the end-to-end delay in IT-WSNs, here we separately analyze the link delay (ld) and node delay (nd) in Equation (1).

**(1) Link Delay:** The link delay comes from the link connection between nodes. In an IT-WSN, due to the change of the environment, $l_{ij}$ is not always valid, and we use $l_{ij}\left(t\right)$ to present the status of link between Nodei and Nodej at discrete time slot *t*. $l_{ij}\left(t\right)$ can be expressed as:

(2) $$l_{ij}\left(t\right)=\left\{\begin{array}{cc} 0 & ,d_{ij}>R\;or\;S_{i}\left(t\right)=0\;or\;S_{j}\left(t\right)=0,\\ 1 & ,d_{ij}\leq R,\;S_{i}\left(t\right)=1\;and\;S_{j}\left(t\right)=1. \end{array}\right.$$

We only evaluate the link quality of $l_{ij}$ at time *t* when $l_{ij}\left(t\right)=1$. To evaluate the link delay caused by $l_{ij}$, we utilize ETX as an indicator. ETX is an indicator for link quality, which is first proposed by De Couto et al. [30], and it is widely utilized in routing path selection, i.e., CTP [16]. The ETX of a link is defined as the expected number of data transmissions required to send a packet over that link (including retransmissions), and the ETX of a route is the sum of the ETX for each link in the route [30]. Here, we take the ETX of a link in Pido. For example, if the ETX of a link is 5, we say the expected transmission times of sending a packet successfully over a link is 5. A smaller value of ETX means better link quality. We define link delay $ld_{ij}\left(t\right)$ as:

(3) $$ld_{ij}\left(t\right)=\lfloor \frac{ETX_{ij}\left(t\right)}{ReTx}\rfloor *T,$$

where ReTx is the limit of retransmissions of the system, *T* is the time interval of system cycle which is also the basic unit of delay, and $ETX_{ij}\left(t\right)$ is the ETX value of $l_{ij}$ at time *t*.

For a network with good link quality, the value of $ETX_{ij}\left(t\right)$ is smaller than that of ReTx generally, i.e., $ld_{ij}\left(t\right)$ is 0, which means a packet can be successfully transmitted within one cycle. However, in an IT-WSN, the value of $ETX_{ij}\left(t\right)$ is often large due to the the presence of tidal water in our system. Specifically, the link quality depicted in Figure 3 shows that the average ETX in the Zhoushan system is 45, which means a packet needs to wait for nine cycles to be sent successfully given ReTx=5. Therefore, $ld_{ij}\left(t\right)$ makes an important part of delays and needs to be taken into consideration for route selection in IT-WSNs.

**(2) Node Delay:** The node delay $nd_{i}\left(t\right)$ is associated with node conditions such as buffer occupancy and node status. For a newly arriving packet, the node delay $nd_{i}\left(t\right)$ is the waiting time for the packet to be sent. $nd_{i}\left(t\right)$ is time-varying and is related to the number of already buffered packets in the node, the processing capability of the current node as well as the node’s future status. The delay of node *i* ($nd_{i}\left(t\right)$) can be defined as:

(4) $$nd_{i}\left(t\right)=\left\{\begin{array}{cc} \frac{B_{i}\left(t\right)}{C_{i}\left(t\right)}*T, & S_{i}^{\prime }\left(t+\tau \right)=1,\\ \infty \;, & S_{i}^{\prime }\left(t+\tau \right)=0, \end{array}\right.$$

where $B_{i}\left(t\right)$ and $C_{i}\left(t\right)$ are the buffer condition and process capability of Nodei at time *t*. Note that nodes in IT-WSN keep generating and storing packets locally during the under-water period, which lead to the number of buffered packets considerable. Both $B_{i}\left(t\right)$ and $C_{i}\left(t\right)$ are time variant.

**Buffer condition ($B_{i}\left(t\right)$):** Buffer condition is the “waiting list” of a node, and can be read from registers. When there are too many packets in the buffer, the node will set a buffer alarm to avoid buffer overflow.

**Processing capability ($C_{i}\left(t\right)$):** $C_{i}\left(t\right)$ is the average process capability during the last *n* periods. We define:

(5) $$C_{i}\left(t\right)=Avg.\left\{C_{i}\left(t-\lambda \right)\right\},\lambda =1,\ldots ,n$$

and we set n=10 in IT-WSN.

**$S_{i}^{\prime }\left(t+\tau \right)$:** In the definition of $nd_{i}\left(t\right)$, we introduce $S_{i}^{\prime }\left(t+\tau \right)$ as the predicted status of Nodei at time t+τ. Here, τ is a parameter that can be set by users, which reveals the tolerance of waiting. If a node is still available at time t+τ, we consider it as a valid neighbor for packet forwarding, otherwise the $nd_{i}\left(t\right)$ will be set as infinite. The average $ld_{ij}\left(t\right)$ is nine cycles in IT-WSN as shown in Figure 3, thus we set τ=10 in our system. We illustrate how to obtain $S_{i}^{\prime }\left(t+\tau \right)$ in Section 4.3.

### 4.3. The Prediction of $S^{\prime }\left(t+\tau \right)$

Predicting $S^{\prime }\left(t+\tau \right)$ accurately is crucial for reducing delay, and a pair of electrodes are embedded on the node to obtain the current node status, which is shown in Figure 1c. When the node is under water, the sea water serves as a conductor and wires two electrodes to makes electrodes conductive. In this way, we can acquire the node status by reading the voltage difference between the two electrodes. However, only the current status (τ=0) can be sensed by electrodes and they are not always precise due to sea waves.

**Figure 6:**
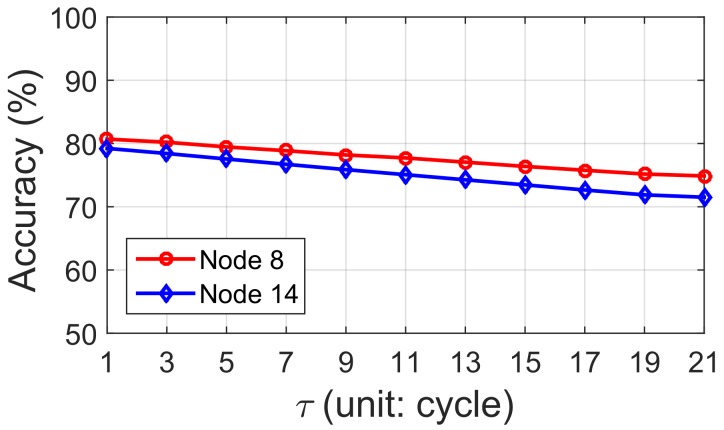
The accuracy of node status prediction with the tidal periodicity.

For a Nodei, obtaining its status directly seems to be impossible when τ>0. Though tidal periodicity can be utilized for roughly predicting node status, the prediction result is not good enough for route selection. To validate our conclusion, we randomly select two nodes (node 8 and node 14) in our Zhoushan system and predict the node status with only tidal periodicity. We compare the prediction status with the real trace we collected in the field test, and results are shown in Figure 6. In Figure 6, the accuracy of predicting node status at the next cycle (τ=1) is only around 80%, and the accuracy is even decreasing for further prediction (τ=2,3…). Remember that nodes only have two states: above-water and under-water, and thus utilizing tidal periodicity alone is not ideal.

To fill in the gap, we utilize a classifier with three indicators to predict node future status, and we describe the details in the following.

#### 4.3.1. Indicators

To improve the prediction accuracy, we propose an indirect method by using implicit indicators which are the above-water duration, the number of available neighbors and the number of available links.

**Above-water duration ($D_{\mathrm{aw}}$):** Though tidal periodicity performs poorly when it is utilized alone, it does provide a rough means of forecasting nodes status. As we assume above, each node maintains a timer $\Delta_{i}$ to record the above-water duration, and we observe the recorded data has correlated periodic cycles. Based on this, we select the above-water duration ($D_{aw}$) as one of the implicit indicators of node status.

**Available neighbors ($N_{\mathrm{nb}}$):** In an IT-WSN, nodes communicate with neighbors frequently to update the routing table. A sudden change of number of neighbors may be caused by the submergence or emergence of neighbors, which reveals the tide rising or falling. Therefore, the change of available neighbors ($N_{nb}$) is also used as an indicator of the future states of nodes.

**Available links ($N_{\mathrm{link}}$):** Link quality is sensitive to environmental changes and can be a sensitive indicator. During the low tide period, the number of available links are stable; during the rising tide period, the physical changes impact link connection. Therefore, the number of available links ($N_{link}$) is a direct indicator of the node status. Note that $N_{nb}$ and $N_{link}$ are different due to the asymmetric links, i.e., an available neighbor does not necessarily hold two available links.

#### 4.3.2. Classifier

For the sake of efficiency, we utilize a classifier for the prediction of $S_{i}^{\prime }\left(t+\tau \right)$, and compare three common classification algorithms: Logistic Regression [31], Ada Boost Classifier [32] and Gaussian Naive Bayesian [33]. Logistic Regression is an implementation-friendly algorithm, which is extremely lightweight in both time and space. Ada Boost Classifier utilizes several weak classifiers to vote for the result, and generally performs well in many classification cases. On the other hand, Ada Boost Classifier is too sensitive to the outliers, which means it may easily lead to over-fitting. Gaussian Naive Bayesian is one of the simplest classification algorithm and can perform well when the data fits Gaussian distribution. The detail of these three algorithms can be referred in [31,32,33]. The comparison of these three algorithms will be stated in Section 4.4. The model is defined as:

(6) $$\begin{array}{c} Model\_1=LogisticRegression(\;L,\;D_{aw},\;N_{nb},\;N_{link}),\\ Model\_2=AdaBoostClassifier(\;L,\;D_{aw},\;N_{nb},\;N_{link}),\\ Model\_3=GaussianNB(\;L,\;D_{aw},\;N_{nb},\;N_{link}). \end{array}$$

Note that what we predict is the future status (τ>0), and we should utilize the future electrode readings as the input. For example, a set of input sample is: $\left\{L\left(t+\tau \right),\;D_{aw}\left(t\right),\;N_{nb}\left(t\right),\;N_{link}\left(t\right)\right\}$.

The mechanism of predicting future status is as follows:

- **Data collection:** We collect training sets from several tide periods at the beginning of the system initialization, and utilize electrode readings (L(t+τ)) as status labels. The indicators can be easily obtained by reading the registers, and the algorithms are feasible to be implemented on WSN sensor nodes.
- **Training a classifier:** We first train each node a specialized classifier at the base station. Though this way can predict future status accurately, we find that it consumes too many calculating resources and storage especially when the network has a large quantity of nodes. Fortunately, the indicators we selected may have similar patterns among nodes. For example, nodes deployed in the same bay will share the same tide period and may have similar wave exposures, and above water duration ($D_{aw}$) may thus also fit similar patterns among nodes. Utilizing this characteristic, we mix the data from all nodes and train a universal classifier.
- **Classifier dissemination:** Base station trains a universal classifier, and broadcasts it to each node. With the classifier and local system knowledge ($D_{aw}\left(t\right),\;N_{nb}\left(t\right),\;N_{link}\left(t\right)$), nodes can predict future status by itself accurately.

### 4.4. Performance Evaluation of Pido

In this section, we evaluate the implementation and performance of Pido with a real-trace-based simulation and compare it with CTP (Collection Tree Protocol) [16] and a delay optimization routing protocol—ORW [10]. As mentioned above, we measure delays in cycles, and the cycle in our IT-WSN is 1 min.

#### 4.4.1. A Comparison between Pido , CTP and ORW

We first provide a brief introduction to CTP and ORW, and then compare the metrics among them. A summary of Pido , CTP and ORW is revealed in Table 2.

**Collection Tree Protocol (CTP)** is a classic routing protocol, which is widely utilized in TinyOS and other embedded operation systems. CTP is designed for WSN with tree topology, and it proposes ETX as routing metric. With continuous link quality estimation, CTP makes nodes to select parents with the least ETX to the sink. In conclusion, routing paths selected by CTP should have optimal expected transmission times, which is energy efficient.

**ORW**, an opportunistic routing protocol, introduces EDC (Expected Duty Cycled wakeups) as routing metric. EDC can be regarded as an adaption of ETX. Besides link quality, EDC also takes the duration of waiting neighbors to be awake into account. ORW strikes a balance between link quality and delay, which should be energy efficient to some extent and can decrease end-to-end delay when compared with CTP.

With the analysis above, CTP should be the most energy-efficient one, and ORW decreases end-to-end delay with the consideration of link quality in the meanwhile. However, both CTP and ORW do not take node status into account, while Pido does. Based on this, in harsh environments like intertidal zones, Pido shows efficiency with lower end-to-end delay.

#### 4.4.2. Simulation Settings

We simulate IT-WSNs in MATLAB (2017a, MathWorks, Natick, MA, USA) with variant network sizes from 49 to 225 (number of nodes) and tidal periods, and we summarize the simulation parameters in Table 3. Nodes are uniformly distributed and the average distance between nodes is 50 m. The base station is located at the center of the network (on the ground, which will not be submerged by water). In the simulation, the maximum communication range of nodes is 150 m, and nodes are allocated with location coordinates $\left(x_{i},y_{i}\right)$. The distance between node *i* and node *j* is calculated by $\sqrt{{\left(x_{i}-x_{j}\right)}^{2}+{\left(y_{i}-y_{j}\right)}^{2}}$. Besides that, we set a countdown timer for each node to record at which time the node will be submerged. To simulate unpredictable waves, we add a strong Gaussian noise at each node.

We implement Pido , CTP and ORW as routing protocols for the simulation. At the beginning of the simulation, each node is buffered with variant number of packets (from 20 to 80). Nodes generate one packet per cycle, and we set the system cycle as 1 min in the simulation. The ETX for CTP, the EDC for ORW and link delay for Pido are calculated by the signal attenuation model with a strong Gaussian noise we mentioned above. To evaluate the energy consumption equally, we set the retransmission times of Pido , CTP and ORW as 10.

#### 4.4.3. Performance Metrics and Simulation Parameters

To evaluate the performance of Pido , we define the performance metrics as follows:

**Delay:** The goal of Pido is to select a path with minimal end-to-end delay. During the evaluation part, we calculate the delay in cycles, which is measured from the time of packet generation until it is received by the sink. In the simulation, the system cycle is 1 min.

**Energy Consumption:** The energy consumption of nodes includes the following: (a) energy consumption during the sleep mode; (b) energy consumption on activating the MCU, sensing the environment during the active mode; and (c) transmitting packets with neighbors during the active mode. Since nodes in IT-WSN work in duty cycle synchronously, we assume that the first two parts of energy consumption are the same for CTP, ORW and Pido . To compare the energy consumption, we focus on measuring the energy for data transmission. We take the average transmission times of a packet during the simulation period as an alternative for energy consumption.

#### 4.4.4. Delay Evaluation

We evaluate Pido in several aspects: delay distribution, delay vs. network size, delay vs. tidal periods and energy consumption. The results are compared with CTP and ORW.

**Figure 7:**
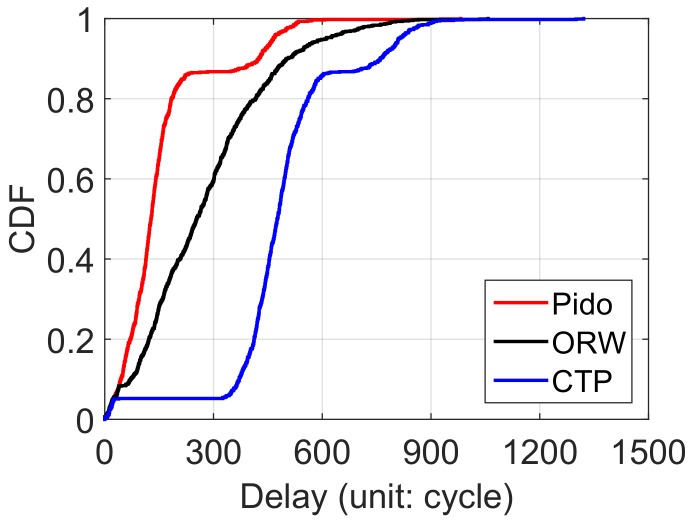
The delay distribution of Pido , ORW and CTP (network size: 225; average under-water time: 360 cycles).

**Delay distribution:** We first compare the system delay distribution among Pido , ORW [10] and CTP [16] in Figure 7. We set the network size as 225 and the average under-water period of each node is 360 cycles (6 h with the system cycle as 1 min). We observe that 80% of delays in Pido is less than 170 cycles while nearly 400 cycles in ORW and 500 cycles in CTP. The result reveals Pido can effectively avoid selecting a submerging node as the next hop with the prediction of nodes future status. We also notice that there is an overlap among CTP, ORW and Pido . This is because one-hop nodes are not affected by the buffer condition and node status, and they can send packets directly to base station. Considering the node status and buffer condition, Pido decreases the system latency in a significant level effectively.

**Figure 8:**
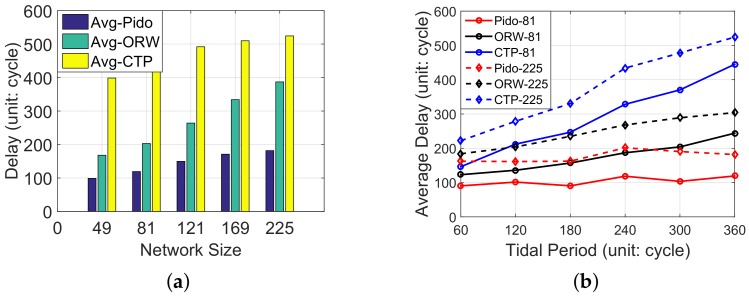
The average delay of Pido , ORW and CTP in different scenarios: network sizes and tidal periods. (**a**) The average delay of Pido , ORW and CTP in different network size (average under-water time: 360 cycles); (**b**) The average delay of Pido , ORW and CTP with different system tidal periods (network sizes are 81 and 225).

**Delay vs. Network sizes:** We then simulate the performance of Pido in different network sizes: 49, 81, 121, 169 and 225. We run the simulation for 10 times as well as CTP and ORW, and the average delay under each network size (average under-water time is set to 360 cycles) is shown in Figure 8a. With the increase of the network size, the average delay is also increased in CTP, ORW and Pido . The reason is that larger scale network also means a longer routing path, so packets will be passed through more hops, which leads to inevitable system delay. Besides this, we also notice Pido decreases an average delay of 69.94% and 43.23% when compared with CTP and ORW among variant network sizes.

**Delay vs. Tidal periods:** To evaluate Pido in variant system tidal periods, we respectively simulate Pido with different average under-water periods for each node and the result is shown in Figure 8b. The result is based on two networks with different sizes: 81 and 225 and we set average disconnection time period between nodes from 60 to 360 cycles. The result in Figure 8b reveals the delay in Pido increases slower when compared with CTP and ORW in different network intermittent characteristics. This is because Pido utilizes more system knowledge and selects paths wisely.

Notably, the average disconnection time in a real IT-WSN ranges from 240 to 360 cycles. During this time period, Pido can save up to 73% delay (69.72% on average) when compared with CTP. Even compared with ORW, a delay optimization routing protocol, Pido still shows better performance in the IT-WSN, which coincides with the analysis in Section 4.4.1. The reason is that ORW is not designed for unpredictable environments like the intertidal zone, and does not take node status into consideration. We also notice when the disconnection time is 240 cycles, the delay in Pido is slightly higher than the 300 and 360 cases, and the delay in CTP also increases faster in this condition. The reason is when the disconnection time is 240 cycles, nodes are more likely to suffer two tidal periods during the transmission process, and the waiting time increases up to double disconnection time (240 × 2), which is even longer than 300 and 360 cycles. In general, Pido reveals robustness in variant system conditions, which indicates Pido can be utilized in intertidal areas with variant tidal periods for data collection.

**Figure 9:**
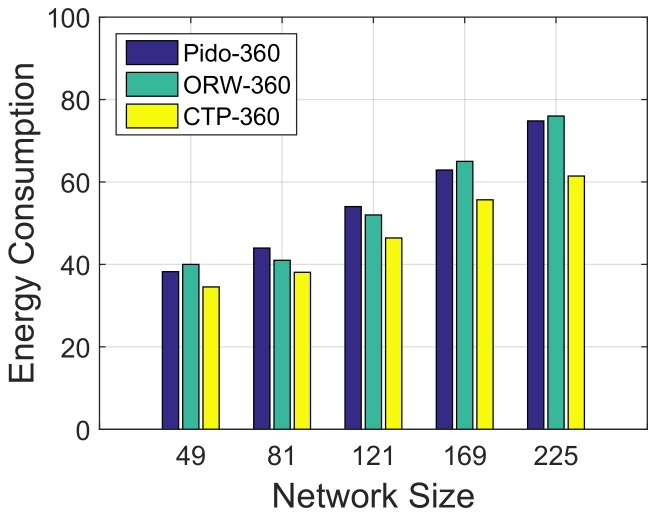
Energy consumption with Pido , ORW and CTP in different network sizes (average under-water time: 360 cycles).

**Energy consumption:** Though Pido decreases the system latency efficiently, inevitable consequence is higher energy consumption than other energy efficient routing protocols. As discussed above, energy consumption during the sleep mode is same with different routing protocols. We set the retransmission limit as 10 for all three algorithms and the energy consumption during the under-water period is also the same. Here, we compare the energy consumption of these three routing protocols by counting the average transmission times of packets during the above-water period in the simulation, and the result is shown in Figure 9. We test energy consumption in variant network sizes from 49 to 225 with a tidal period of 360 cycles. In Figure 9, the energy consumption of Pido and ORW are similar, and are higher than CTP, which is consistent with the analytical results in Section 4.4.1. The additional energy consumption increases with an average of 15.47% when comparing Pido with CTP (10.73% with a network size of 49 and 21.77% with a network size of 225). Recall that, in Figure 8b, when system tidal period is 360 cycles, Pido can save up to 73% average delay when the network size is 225. Based on this, we consider the additional energy consumed by Pido is acceptable in a delay-priority system.

#### 4.4.5. A Comparison of the Field Test and the Simulation

To further evaluate the performance of Pido , we validate the effectiveness of Pido with and without using it in the field test as well as in the simulation, and the results are summarized in Table 4. We deployed 27 nodes in the field test, and the average under-water time at intertidal zone in Zhoushan is 458 cycles. Therefore, we set the network size and the average under-water time as 27 nodes and 458 cycles in the simulation accordingly. As shown in Table 4, the average delay decreases significantly when Pido is implemented in the field test. We also notice that the field test reveals longer average delay and higher energy consumption than the simulation results. The reason is that the environment is much uncontrollable in the field test, which leads to the link quality being even worse in the field test. On the other hand, different distributions of nodes may also lead to different routing paths in the simulation and the field test. However, the results in the field test with Pido reveal that Pido can decrease the system latency efficiently.

## 5. Assessment of **Pido**

In this section, we conduct an overall assessment of Pido . We first assess the delay decreased by Pido with a node status classifier, and we then evaluate the accuracies of the classifiers with different algorithms.

### The Delay Distribution of Pido

**Figure 10:**
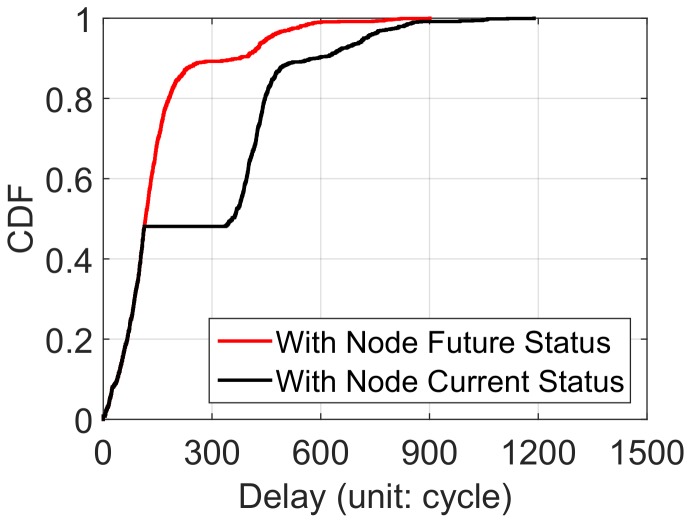
The delay distribution of Pido with future node status and current node status (network size: 225; average under-water time: 360 cycles).

We compare the delay distribution with the knowledge of node future status (with classifier) and current status (with electrodes) in Figure 10. We design a classifier to predict the nodes future status, which can be utilized for routing selection in Pido . As above, a pair of electrodes is also embedded in our sensor nodes to detect submersion. We observe that nearly half of the delays are the same between these two mechanisms. The reason is that they choose the same routing paths when nodes are truly above water according to the delay metric proposed in Pido . However, Figure 10 also reveals that nodes are able to select routing paths more wisely and avoid being trapped in submerging nodes with the guidance of the node future status. The result in Figure 10 demonstrates that Pido with the classifier can decrease the system latency efficiently.

#### The Evaluation of Predicting $S^{\prime }\left(t+\tau \right)$

**Figure 11:**
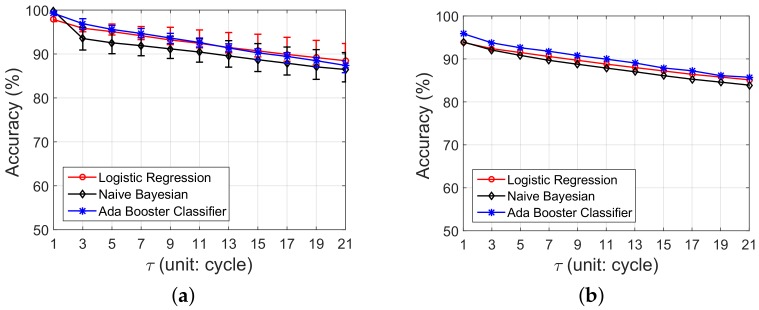
The prediction accuracy of node status with three classification algorithms: Logistic Regression, Naive Bayesian and Ada Booster Classifier. (**a**) Training classifiers for each node with three classification algorithms; (**b**) Training a universal classifier for the network with three classification algorithms.

As mentioned in Section 4.2, $S^{\prime }\left(t+\tau \right)$ is the prediction of node status at time t+τ. We utilize three indicators: above-water duration ($D_{aw}$), available neighbors ($N_{nb}$) and available links ($N_{link}$) at time *t* to predict node status at time t+τ. Three algorithms that include Logistic Regression, Ada Boost Classifier and Gaussian Naive Bayesian are attempted in Pido . To validate which algorithm is more suitable for the IT-WSN, we compare the performance of these three algorithms based on the field test results in the Zhoushan system, and the results can be referred to in Figure 11.

**Each node has a unique classifier:** A straightforward idea is training each node with a unique classifier. We utilize real trace data from the IT-WSN in Zhoushan and evaluate the prediction accuracy of three algorithms with τ from 1 to 21 cycles. The prediction accuracy of node status in the IT-WSN is shown in Figure 11a. We find that the prediction accuracy decreases with the increase of τ. The reason is that the features we selected for status prediction are time sensitive, which means they are more valid when utilized for a comparatively shorter time prediction. When τ=1, all three of these classifiers can reach a high prediction accuracy over 97%, which reveals that the indicators we select are strongly related to the node status. Recall that, in Figure 6, we utilize a tidal predictor to predict nodes’ future status. Our classifier can increase around 17% for each τ when compared with the results in Figure 6. On the other hand, we also notice that the prediction accuracy of Logistic Regression decreases slower than the other two algorithms, and even performs better when τ>13.

**A single classifier for all nodes:** Training specialized classifiers for nodes can reach a good performance, but it also leads to huge overhead on both dissemination and calculation. To solve this, we attempt to find a universal classifier for all nodes. We utilize real trace with Logistic Regression, Ada Boost Classifier and Gaussian Naive Bayesian to train the universal classifier. The results are shown in Figure 11b. With the increase of τ, the prediction accuracy also decreases for each algorithm. Though the prediction accuracy is not so good as in Figure 11a, both Ada Boost Classifier and Logistic Regression perform a prediction accuracy over 85% when τ=21, which is acceptable for Pido . It is worth mentioning that Logistic Regression introduces less overhead on both training time and storage than Ada Boost Classifiers, which are sensitive for embedded sensors. Taking all the factors into consideration, we choose Logistic Regression in Pido .

## 6. Conclusions

This paper presents Pido, a predictive delay optimization route selection mechanism for intertidal wireless sensor networks. Based on our observations in the field test, IT-WSN suffers from poor link quality and dynamic connections, which lead to intolerable delays. Pido considers delay introduced both by the link and the node comprehensively, which decreases the end-to-end delay efficiently with low overhead. Pido utilizes implicit system indicators to predict nodes future status accurately, and helps nodes to select routes wisely. Extensive evaluation is conducted in both real systems and large-scale simulation. The results indicate that Pido can decrease end-to-end delay up to 73% with little overhead.

## Figures and Tables

**Table 1:** Notations.

$d_{ij}$	The physical distance between Nodei and Nodej.
R	Transmission range.
$l_{ij}$	Physical connection between Nodei and Nodej.
$ETX_{ij}\left(t\right)$	The expected transmission times of $l_{ij}$.
$ld_{ij}\left(t\right)$	Delay introduced by $link_{ij}$ at time *t*
$nd_{i}\left(t\right)$	Delay introduced by Nodei at time *t*
$S_{i}\left(t\right)$	Current status of Nodei at time *t*; Nodei is available at time *t* if $S_{i}\left(t\right)=1$ and vice versa.
$S_{i}^{\prime }\left(t+\tau \right)$	The predicted status of Nodei at time t+τ.
$B_{i}\left(t\right)$	The number of packets buffered in Nodei at time *t*.
$C_{i}\left(t\right)$	The number of packets can be sent by Nodei at time *t* in a cycle.
ReTx	The limit of retransmission times in a cycle.
*T*	The time interval of a system cycle. The basic time unit of delay.

**Table 2:** The comparison between Pido , CTP and ORW.

Protocol	Metric	The Calculation of Metric
Pido	delay	$delay=\sum_{i=1}^{n}ld_{i,i+1}+\sum_{j=2}^{n-1}nd_{j+1}$
CTP	ETX (Expected Transmissions)	Calculated by a Link Quality Estimator
ORW	EDC (Expected Duty Cycled wakeups)	$EDC_{i}\left(S_{i}\right)=\frac{1}{\sum_{j\in S_{i}}p_{ij}}+\frac{\sum_{j\in S_{i}}\in S_{i}p_{ij}EDC_{j}}{\sum_{j\in S_{i}}\in S_{i}p_{ij}}+\omega$ *

* definitions of $S_{i}$, $p_{ij}$ and ω can be referred in [10].

**Table 3:** The summary of the simulation parameters.

Parameters	Description
Network Size	The number of nodes in the network. In our simulation, we set the network sizes vary from 49, 81, 121, 169 and 225.
Tidal Period	The time duration of a tidal period. In our simulation, we set the tidal period vary from 60, 120, 180, 240, 300, 360 cycles.

**Table 4:** The comparison of the field test results and simulation results.

	Network Size	Average Under-Water Time	Average Delay (Cycle) ^1^	Energy Consumption ^2^
Field Test with Pido	27 nodes	458 cycles	93.9	35.6
Field Test without Pido	27 nodes	458 cycles	374	37
Simulation	27 nodes	458 cycles	72.8	32.2

^1^ The average delay is calculated in cycles. ^2^ The energy consumption is calculated by counting the average transmission times of packets.

## References

[1] Helmuth B., Mieszkowska N., Moore P., Hawkins S.J. (2006). Living on the edge of two changing worlds: Forecasting the responses of rocky intertidal ecosystems to climate change. Annu. Rev. Ecol. Evol. Syst..

[2] Helmuth B. (2002). How do we measure the environment? Linking intertidal thermal physiology and ecology through biophysics. Integr. Comp. Biol..

[3] Helmuth B., Choi F., Matzelle A., Torossian J.L., Morello S.L., Mislan K., Yamane L., Strickland D., Szathmary P.L., Gilman S.E. (2016). Long-term, high frequency in situ measurements of intertidal mussel bed temperatures using biomimetic sensors. Sci. Data.

[4] Xu M., Xu W. Taco: Temperature-aware compensation for time synchronization in wireless sensor networks. Proceedings of the 2013 IEEE 10th International Conference on Mobile Ad-Hoc and Sensor Systems (MASS).

[5] Xu M., Xu W., Han T., Lin Z. (2016). Energy-efficient time synchronization in wireless sensor networks via temperature-aware compensation. ACM Trans. Sens. Netw. (TOSN).

[6] Little T.D., Konrad J., Ishwar P. A wireless video sensor network for autonomous coastal sensing. Proceedings of the Conference on Coastal Environmental Sensing Networks (CESN 2007).

[7] Wang X., Wang X., Xing G., Yao Y. Dynamic duty cycle control for end-to-end delay guarantees in wireless sensor networks. Proceedings of the 2010 IEEE 18th International Workshop on Quality of Service (IWQoS).

[8] Yao Y., Cao Q., Vasilakos A.V. (2015). EDAL: An energy-efficient, delay-aware, and lifetime-balancing data collection protocol for heterogeneous wireless sensor networks. IEEE/ACM Trans. Netw. (TON).

[9] Dong M., Ota K., Liu A., Guo M. (2016). Joint optimization of lifetime and transport delay under reliability constraint wireless sensor networks. IEEE Trans. Parallel Distrib. Syst..

[10] Landsiedel O., Ghadimi E., Duquennoy S., Johansson M. Low power, low delay: Opportunistic routing meets duty cycling. Proceedings of the 11th International Conference on Information Processing in Sensor Networks.

[11] Srinivasa S., Krishnamurthy S. CREST: An opportunistic forwarding protocol based on conditional residual time. Proceedings of the 2009 6th Annual IEEE Communications Society Conference on Sensor, Mesh and Ad Hoc Communications and Networks.

[12] Bulut E., Geyik S.C., Szymanski B.K. Conditional shortest path routing in delay tolerant networks. Proceedings of the 2010 IEEE International Symposium on “A World of Wireless, Mobile and Multimedia Networks” (WoWMoM).

[13] Srinivasa K., Levis P. RSSI is under Appreciated [C/OL]. Proceedings of the 3rd Workshop on Embedded Networded Sensors (EmNets 2006).

[14] Ganesh S., Amutha R. (2013). Efficient and secure routing protocol for wireless sensor networks through SNR based dynamic clustering mechanisms. J. Commun. Netw..

[15] Liang J.J., Yuan Z.W., Lei J.J., Kwon G.I. Reliable routing algorithm on wireless sensor network. Proceedings of the 2010 The 12th International Conference on Advanced Communication Technology (ICACT).

[16] Gnawali O., Fonseca R., Jamieson K., Moss D., Levis P. Collection tree protocol. Proceedings of the ACM SenSys.

[17] Fonseca R., Gnawali O., Jamieson K., Levis P. Four-Bit Wireless Link Estimation. Proceedings of the ACM HotNets-VI.

[18] Jakllari G., Eidenbenz S., Hengartner N., Krishnamurthy S.V., Faloutsos M. (2012). Link positions matter: A noncommutative routing metric for wireless mesh networks. IEEE Trans. Mob. Comput..

[19] Zhang H., Sang L., Arora A. (2010). Comparison of data-driven link estimation methods in low-power wireless networks. IEEE Trans. Mob. Comput..

[20] Liu M., Cao J., Chen G., Wang X. (2009). An energy-aware routing protocol in wireless sensor networks. Sensors.

[21] Hassanein H., Luo J. Reliable energy aware routing in wireless sensor networks. Proceedings of the 2006 Second IEEE Workshop on IEEE Dependability and Security in Sensor Networks and Systems.

[22] Moeller S., Sridharan A., Krishnamachari B., Gnawali O. Routing without routes: The backpressure collection protocol. Proceedings of the 9th ACM/IEEE International Conference on Information Processing in Sensor Networks.

[23] Paine R.T., Fenchel T., Kinne O. (1994). Marine Rocky Shores and Community Ecology: An Experimentalist’s Perspective.

[24] Kish N.E., Helmuth B., Wethey D.S. (2016). Physiologically grounded metrics of model skill: A case study estimating heat stress in intertidal populations. Conserv. Physiol..

[25] Lima F.P., Burnett N.P., Helmuth B., Kish N., Aveni-Deforge K., Wethey D.S. (2011). Monitoring the Intertidal Environment with Biomimetic Devices.

[26] Helmuth B., Broitman B.R., Yamane L., Gilman S.E., Mach K., Mislan K.A.S., Denny M.W., Barnes B., Gordon M., Sato K. (2010). Organismal climatology: Analyzing environmental variability at scales relevant to physiological stress. J. Exp. Biol..

[27] Juang P., Oki H., Wang Y., Martonosi M., Peh L.S., Rubenstein D. (2002). Energy-efficient computing for wildlife tracking: Design tradeoffs and early experiences with ZebraNet. ACM Sigplan Not..

[28] Liu Y., He Y., Li M., Wang J., Liu K., Li X. (2013). Does wireless sensor network scale? A measurement study on GreenOrbs. IEEE Trans. Parallel Distrib. Syst..

[29] Liu G., Tan R., Zhou R., Xing G., Song W.Z., Lees J.M. Volcanic earthquake timing using wireless sensor networks. Proceedings of the ACM IPSN.

[30] De Couto D.S., Aguayo D., Bicket J., Morris R. (2005). A high-throughput path metric for multi-hop wireless routing. Wirel. Netw..

[31] Walker S.H., Duncan D.B. (1967). Estimation of the probability of an event as a function of several independent variables. Biometrika.

[32] Freund Y., Schapire R.E. (1995). A desicion-theoretic generalization of on-line learning and an application to boosting. Proceedings of the European Conference on Computational Learning Theory.

[33] John G.H., Langley P. (1995). Estimating continuous distributions in Bayesian classifiers. Proceedings of the Eleventh Conference on Uncertainty in Artificial Intelligence, Montreal, QU, Canada, 18–20 August 1995.

